# Intramedullary spinal cord metastasis from laryngeal carcinoma: case report and review of literature

**DOI:** 10.11604/pamj.2017.26.189.11507

**Published:** 2017-03-30

**Authors:** Mohamed Sahli, Bouchaib Hemmaoui, Fouad Benariba

**Affiliations:** 1 Department of Head and Neck Surgery, Military Hospital Mohammed V, Rabat, Morocco

**Keywords:** Larynx, squamous cell carcinoma, intramedullary spinal cord metastasis

## Abstract

Laryngeal cancer metastases are relatively rare and mainly affect the lung. The medullary localization remains exceptional. We report the case of a patient followed for operated laryngeal cancer and whose oncologic control revealed a medullary localization. A patient followed for squamous cell carcinoma of the larynx, treated in 2010 by a partial surgery whose endoscopic control at 5 years revealed the presence of right arytenoid edema without suspicious lesions, multiple biopsies were made and which returned negative. A month later, the patient presented a rebel cervical spine pain and a feeling of heaviness of the upper limbs, for which a radiological assessment was done finally objectifying a right hypopharyngeal process and a suspicious right internal jugular lymphadenopathy (biopsy confirmed the squamous type), as well as an intramedullary metastasis. This case is an illustration of an exceptional evolution of this type of cancer and a are metastatic localization difficult to highlight, which leads us to ask the question on the need of simultaneous and systematic radiological and endoscopic control treatment for operated laryngeal cancer.

## Introduction

Intramedullary spinal cord metastasis (ISCM) represents an extremely rare complication [1]. Cancers of the head and neck rarely give this type of metastasis; it is usually a nasopharyngeal cancer [2]. We report a case of intramedullary metastasis of squamous cell carcinoma of the larynx in order to raise the diagnostic and therapeutic difficulties with review of the literature.

## Patient and observation

**Figure 1:**
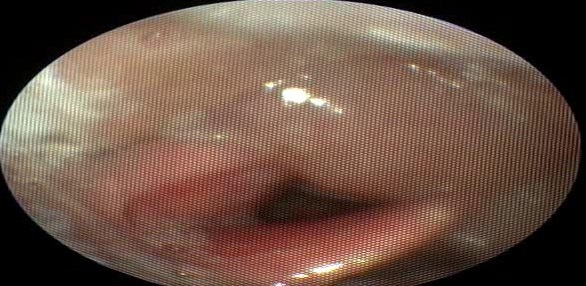
Carcinological control endoscopy showing a right arytenoid edematous aspect filling the homolateral piriform sinus without suspect lesion

**Figure 2:**
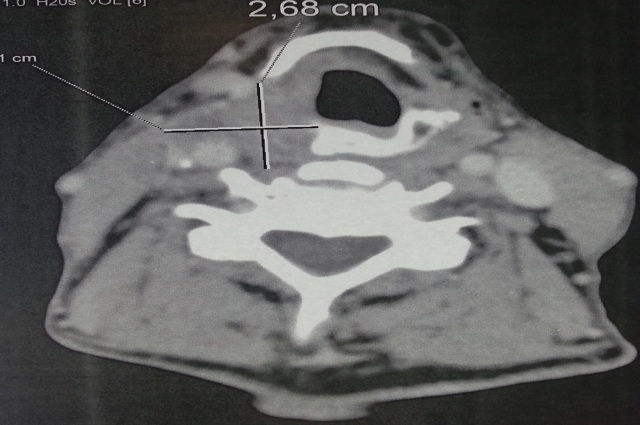
A cervical tomodensitometry showing a right hypopharyngeal process measuring 30 × 27mm coming into contact with the right primitive carotid artery

**Figure 3:**
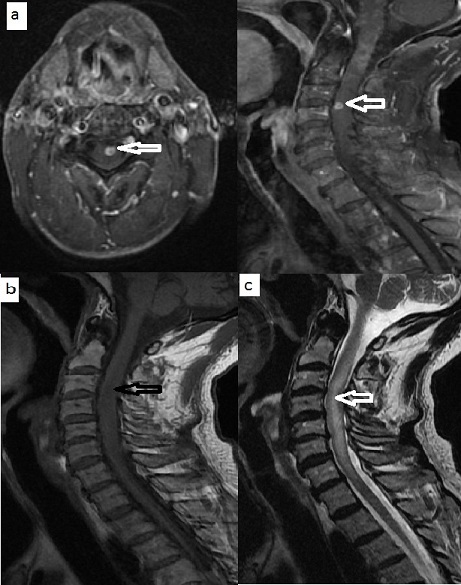
A) sagittal MRI and axial T1 sequence with gadolinium injection: annular contrast enhancement zone at the anterior portion of the cervical spinal cord opposite C4-C5 (arrow); B) Sagittal MRI T1 sequence: isointense nodule (arrow); C) Sagittal MRI T2 sequence: fusiform aspect of the spinal cord resulting in an intramedullary edema (arrow)

A 68-year-old patient, operated in 2010 for squamous cell carcinoma of the larynx classified T1bN0M0, he received a partial laryngectomy with crico-hyoid-epiglotto-pexie (CHEP). The suites were simple, the resection margins were negative but with a poorly differentiated character and perineural invasion on histological examination. Regular control was unremarkable. Five years later, the patient presented progressive dyspnea, the endoscopic examination of the larynx showed a right arytenoid edema filling the homolateral piriform sinus with no suspicious lesion (Figure 1). A cervical scanner has objectified a right hypopharyngeal process coming in contact with the right primary carotid artery (Figure 2); multiple biopsies performed twice were negative. A month later, the patient a rebel cervical spine pain and a heaviness of the upper limbs, in front of which a cerebro-cervical magnetic resonance imaging (MRI) was requested objectifying a right hypopharyngeal process, suspicious right internal jugular lymphadenopathy (level III) of about 1cm, with a cocardial nodule of the cervical spinal cord taking the contrast evoking secondary localizations (Figure 3). Cervicotomy was made and the histological result was in favor of poorly differentiated squamous cell carcinoma. The Positron Emission Tomography–Computed Tomography (PET- CT) requested in staging confirmed the medullary localization, with no other secondary sites. Cervical spinal cord radiotherapy (30Gy in 10 fractions) associated with corticosteroid therapy was started followed by palliative chemotherapy with 5-fluorouracil and docetaxel. The patient died three months after the diagnosis of MIM.

## Discussion

ISCM in general are rare; they account for 4.2% to 8.5% of the metastases of the central nervous system, the primary tumor is often localized in the lung (48%) followed by breast (16%) [1]. Metastases of laryngeal cancer are relatively rare, occurring in about 19% of cases [3]. Medullary location is exceptional, and to our knowledge only two cases have been reported in the literature (Table 1) [4, 5]. Three modes of dissemination are possible explaining these metastatic localizations: hematogenic, by contiguity, or by carcinomatous meningitis [6, 7]. The single cervical spinal cord metastatic localization in our patient advocates in favor of a spread by contiguity rather than hematogenous dissemination. MRI represents the examination of choice in case of suspicion of ISCM. The T2 sequences show a hyperintensity reflecting the existence of a tumor infiltration. After injection of gadolinium, the contrast is frank, nodular or annular, sometimes surrounded by a hypo-intensity related to peritumoral edema [1, 7]. The clinical and metastatic context, the radiological aspect and the chronology of the events allowed us to retain the diagnosis of ISCM in our patient. The poorly differentiated character and the presence of perineural invasion are the most prognostic factors reported in the literature [2, 8]. These predictive elements were reported in the initial pathologic examination in our patient explaining the evolution and aggressiveness of his disease. It is recognized that local recurrence favors lymph node recurrence, which in turn promotes the development of metastases. The majority of authors insist that recurrences are more often detected by complementary examinations in asymptomatic patients [9]. In our patient, the imaging allowed us to detect suspicious internal jugular lymphadenopathy and confirmed histologically, whereas the clinical examination had not demonstrated palpable lymphadenopathy. There is currently no consensus regarding the treatment of these recurrences with ISCM [1]. Radiotherapy with or without corticosteroids is the most widely reported protocol in the literature. Chemotherapy is used as adjuvant therapy for multiple metastases. Surgery is reserved for radio-resistant ISCM or ISCM unique limited well without other secondary locations, it allows a stabilization or regression of neurological disorders, and an improvement in overall survival [1, 10].

**Table 1:** Characteristics of ISCM cases of laryngeal cancer described in the literature

	Age /sexe	clinical signs	Diagnostic delay	Location	Overall survival after diagnosis of metastasis
**Dağli et al [4]. 2007**	40/Man	Spinal pain / numbness of the lower limbs	Concomitant with the diagnosis of laryngeal cancer	Dorsal	10 days
**Mendes et al [5]. 2004**	60/Man	Left shoulder pain / motor deficit of the upper limbs	20 months	Cervical (C5)	6 weeks

## Conclusion

ISCMs of laryngeal cancer are exceptional. Our case illustrates the difficulty of diagnosis of recurrence based on a simple physical examination, and the contribution of MRI in the detection of these metastases. The therapeutic attitude is controversial due to the lack of controlled studies on the effectiveness of different approaches. The most important predictor of functional outcome is certainly neurological status; therefore, early diagnosis is the key to improve the prognosis.
